# Neutrality and the Response of Rare Species to Environmental Variance

**DOI:** 10.1371/journal.pone.0002777

**Published:** 2008-07-23

**Authors:** Lisandro Benedetti-Cecchi, Iacopo Bertocci, Stefano Vaselli, Elena Maggi, Fabio Bulleri

**Affiliations:** 1 Dipartimento di Biologia, Università di Pisa, Pisa, Italy; 2 Laboratory of Coastal Biodiversity, Centro Interdisciplinar de Investigação Marinha e Ambiental, University of Port, Port, Portugal; Centre National de la Recherche Scientifique, France

## Abstract

Neutral models and differential responses of species to environmental heterogeneity offer complementary explanations of species abundance distribution and dynamics. Under what circumstances one model prevails over the other is still a matter of debate. We show that the decay of similarity over time in rocky seashore assemblages of algae and invertebrates sampled over a period of 16 years was consistent with the predictions of a stochastic model of ecological drift at time scales larger than 2 years, but not at time scales between 3 and 24 months when similarity was quantified with an index that reflected changes in abundance of rare species. A field experiment was performed to examine whether assemblages responded neutrally or non-neutrally to changes in temporal variance of disturbance. The experimental results did not reject neutrality, but identified a positive effect of intermediate levels of environmental heterogeneity on the abundance of rare species. This effect translated into a marked decrease in the characteristic time scale of species turnover, highlighting the role of rare species in driving assemblage dynamics in fluctuating environments.

## Introduction

Explaining the causes of variation in patterns of distribution and abundance of species within and between habitats has proved to be a formidable task for ecologists, due to the complexity of ecological systems [1]. Hubbell's neutral theory of biodiversity [2] has renewed the interest over two long-standing views of assemblage dynamics that contrast deterministic versus stochastic processes [1], [3]. Deterministic models emphasize differences in species' life histories that translate into differential competitive abilities and resource requirements [4]–[6]. Stochastic models stress that changes in assemblages result predominantly from random variation in the fundamental biological processes of birth, death and migration [2], [7], so that differences among species in life-history traits may be neglected. It is important to note that whilst the neutral theory of biodiversity is a stochastic theory and it assumes that species have the same rates of death and reproduction on a per capita basis [2], stochastic models do not necessarily negate species differences. As shown by recent theoretical work [8], stochastic processes can be introduced into deterministic competitive models, bridging a gap between classic niche theory and neutral views of assemblage dynamics.

Neutrality implies that environmental heterogeneity does not translate into differential probabilities of success for individual species [2], [9]. Under neutrality, stochastic events of death and colonization of individuals coupled with dispersal limitation result in ecological drift – i.e. autocorrelated patterns of species abundances and assemblage turnover in space and time. Ecological drift has provided a benchmark for empirical tests of neutrality based on observational data [3], [7], [10], [11].

Ecological drift is not the only mechanism that can explain gradual changes in composition and abundance of species in space and time. The differential response of species to environmental heterogeneity can also produce autocorrelated patterns of variation in assemblages [12]–[14]. Distinguishing between these alternatives requires coupling correlative analyses of assemblage dynamics over large spatial and temporal scales with more focused experimental tests of the influence of environmental variance on species abundance distributions and dynamics [3], [14], [15].

Correlative studies have a central role in tests of neutrality and novel analytical techniques are continuously developed to enable the fit of neutral and non-neutral models to observational data [16]–[19]. A causal understanding of the mechanisms underlying assemblage dynamics, however, requires manipulative experiments. To our knowledge, only two studies have provided experimental tests of the neutral theory and both have provided evidence against it [6], [20]. Because neutral and non neutral dynamics may be seen as the two extremes of a continuum of explanatory models, integrating deterministic and stochastic views into a synthetic theory seems a more sensible approach to improve our understanding of assemblage dynamics than maintaining conceptual dichotomies [11], [21]–[26]. To achieve this integration it is necessary to understand at what scales and for which taxa the assumption of ecological equivalence holds.

Here we combine a study of temporal change in assemblages of algae and invertebrates of rocky seashores over a period of 16 years, with an experimental analysis of the influence of temporal variability of disturbance on these assemblages. We use the observational data to test the prediction that observed temporal patterns of decay in similarity would be undistinguishable from those generated by a neutral model based on ecological drift under realistic patterns of natural disturbance, if neutrality holds. With the experimental data we test the hypothesis that, under neutrality, assemblages would not be affected by changes in temporal variance of disturbance (the clustering of events in time), provided that overall intensity of disturbance (i.e. the total number of events and their magnitude over a given period of time) does not change with levels of variance. This hypothesis is based on the argument that neutral assemblage dynamics depend on death and colonization events that are a function of the number of individuals removed by disturbance, in addition to dispersal limitation. Thus, in a series of disturbances, it is the overall intensity of events that should matter, not the temporal variance of the series [15]. However, the extent to which neutrality can be tested by examining assemblage responses to environmental stochasticity is unclear [27]. We substantiate our conjecture by showing with simulations that temporal variance of disturbance has no effect on species rank-abundance curves under neutrality, either under constant or variable rates of immigration [28]. Furthermore, we consider that temporal variance of disturbance elicits non-neutral responses only if it modifies species abundance distributions by influencing the probability of occurrence of individual species or groups of species, consistently with the definition of neutrality.

To test whether the response of assemblages to changes in environmental variance is consistent with neutrality or deviates from it, we fit to the experimental data both the dispersal unlimited and dispersal limited versions of the neutral theory of biodiversity [2], reflecting meta and local communities, respectively. We consider both models because the distinction between large and local patterns of species abundance based on dispersal becomes artificial in continuous landscapes [28], so we have no *a priori* expectation of which, if any, of the two models would be the most appropriate for our assemblages. If neutrality holds, however, temporal variance of disturbance would have no effect on species abundance data, so the outcome of the comparison of the two models should be the same across experimental levels of temporal variance of disturbance.

Changes in temporal variance of disturbance may have long-term effects on assemblage dynamics if they affect the probability of occurrence of species. Theoretical studies and laboratory experiments have indicated that negative density-dependence in population growth may reduce the risk of extinction and increase population abundance of rare species during long periods of adverse environmental conditions, which are more likely to occur under high levels of environmental variance [14], [29], [30]. Analytical treatments have also shown that the magnitude of density-dependence processes relative to demographic stochasticity is an important determinant of the temporal dynamics of an ecological system [31]. We apply the procedures outlined by Azaele et al. [31] to the experimental data to test whether environmental fluctuations increase assemblage turnover, as it would be expected if negative density-dependent effects operated in the system. Density-dependent regulation would enable depauperated populations to recover quickly after a disturbance until another disturbance resets abundances to low values. Increasing the temporal variance of disturbance would therefore lead to larger temporal fluctuations in species abundance compared to circumstances in which disturbances were distributed more regularly in time or in the absence of density-dependent regulation.

## Results

### Observational data: temporal changes in assemblages

**Figure 1:**
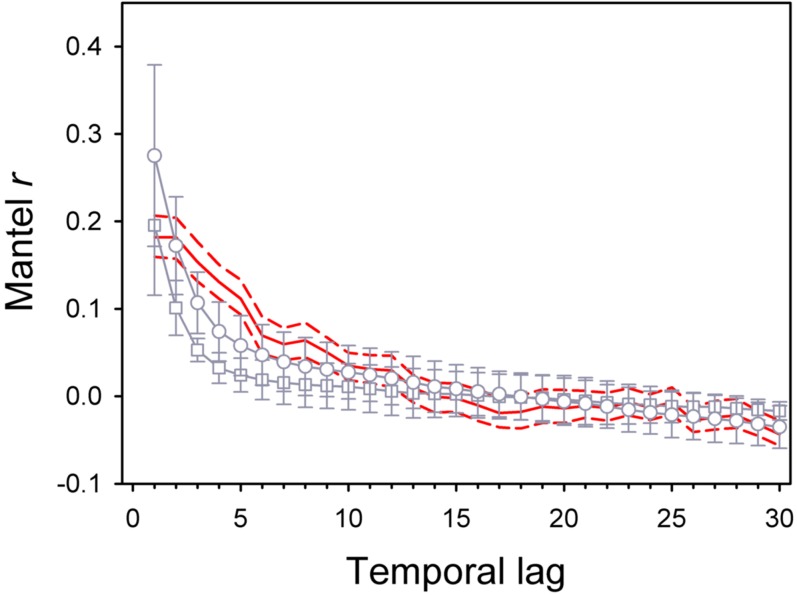
Comparison of observed and expected temporal patterns of decay of similarity. Data are for assemblages of rocky shores (continuous red line) and assemblages generated under ecological drift (grey lines and symbols) with seasonal variation in disturbance. A temporal lag corresponds to a period of three months. Mantel r's were based on the Bray-Curtis (BC, circles) and Squared Cord Distance (SCD, squares) indexes of similarity for modeled data. Only temporal patterns based on BC similarities are shown for observed data; those based on the SCD index were similar. Dashed red lines are 95% Confidence Intervals obtained by bootstrapping the empirical data 1000 times with replacement. Error bars for modeled data are 1 standard deviation over 100 replicated simulations. Mantel's coefficients for observed data were significantly different from zero (unadjusted probabilities) from lag 1 to lag 13 and from lag 20 to lag 30. Model parameters were estimated from a data set containing 29 species and were θ = 4.71 and *m* = 0.017; mortality was distributed evenly across time steps and corresponded to one turnover of the assemblage over the course of the study (see Figure S1 for other disturbance scenarios). Observed patterns based on the SCD index of similarity deviated more from expectation that those based on the BC index from lag 2 to lag 8.

A total of 68 species were identified over the course of the study. Analyses that focused on the most abundant species [using the Bray-Curtis index, see eq. (1) in Materials and Methods], revealed a good correspondence between observed and modeled data with two possible exceptions occurring at the smallest time scale (3 months), where the neutral model predicted higher (but uncertain) correlation compared to observed data, and between time lags 4 and 5, where the model slightly underestimated observed correlations (Figure 1). A very similar result was obtained using the Jaccard index of similarity [eq. (2) in Materials and Methods], which emphasized compositional changes in assemblages (Figure S1). In contrast, analyses based on the Squared Cord Distance index [eq. (3) in Materials and Methods], which emphasized differences in abundance of rare species, indicated that the neutral model estimated temporal autocorrelation correctly at the shortest time scale and at time scales larger than 24 months (from time lag 8 onwards), while it underestimated autocorrelation between 3 and 24 months (Figure 1). For this period, Mantel's coefficients derived from simulated data were outside the 95% confidence intervals for the coefficients obtained from the observed data. The level of underestimation increased with increasing intensity of disturbance for all indices (Figure S1).

### Experimental data: effects of temporal variance of disturbance

Temporal variance of disturbance had no effect on species rank-abundance distributions resulting from neutral drift, either under constant or variable rates of immigration (Figure S2). This outcome supported our original hypothesis that neutral assemblages would not be affected by changes in variance of disturbance, provided that intensity is kept constant across levels of variance.

Analysis of the experimental data proceeded with the comparison of the fit of the local Zero-Sum-Multinomial (lZSM) and meta Zero-Sum-Multinomial (mZSM) distributions to the Control, low-variance (LV), medium-variance (MV) and high-variance (HV) conditions, separately. These analyses were based on the likelihood-ratio statistic (LR), as it is commonly done to compare the fit of different models to a given data set. It is important to note, however, that the LR statistic is negatively related to species richness under a true null hypothesis of neutrality – i.e. the power of the test depends on species richness (Jabot and Chave, unpublished data). Thus, the canonical approach to assess the significance of the LR statistic using the *χ* ^2^ distribution is not appropriate when the goal is to compare the outcome of multiple tests performed across assemblages that differ in species richness, as in our case. To circumvent this problem, we assessed the significance of the LR statistic by comparing observed values to null distributions derived by simulating neutral drift under the same levels of richness as those observed in the Control, LV, MV and HV conditions and under realistic regimes of disturbance (see Materials and Methods for further details; the distributions are reported in Figure S3). These tests indicated that the lZSM did not provide a better fit than the mZSM to the Control data and to any of the experimentally disturbed assemblages (Table 1). Thus, the same distribution fitted species abundance data originated under different levels of temporal variance of disturbance, so that neutrality was not rejected by these tests.

**Table 1:** Parameter estimates and test statistics for experimental data.

	Total abundance	Richness	mZSM	lZSM		LR	*P*
Parameters	*J*	*S*	θ	θ	*m*		
Control	4597	32	4.9	7.7	0.039	2.1	0.25
LV	3279	28	4.1	5.5	0.094	2.4	0.553
MV	3398	28	4.1	5.2	0.069	6.1	0.135
HV	3490	25	3.6	5.1	0.059	6.0	0.098

LR, Likelihood Ratio test with probabilities (*P*) obtained from null distributions of the statistic (Figure S3; see text for further details); mZSM, meta Zero-Sum-Multinomial-Distribution; lZSM, local Zero-Sum-Multinomial distribution; LV, Low Variance; MV, Medium Variance; HV, High Variance.

**Figure 2:**
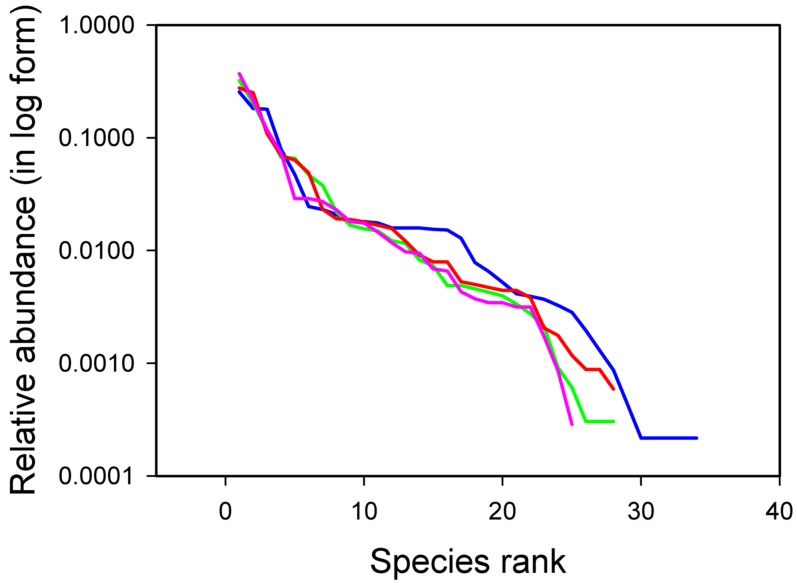
Rank-abundance distributions. Data are for Control (blue line), Low Variance (green dashed line), Medium Variance (red line) and High Variance (pink line) experimental conditions. The Control and Low Variance curves differed from the Medium and High Variance curves for the presence of a tail of rare species. Full statistics are reported in Table 1.

**Figure 3:**
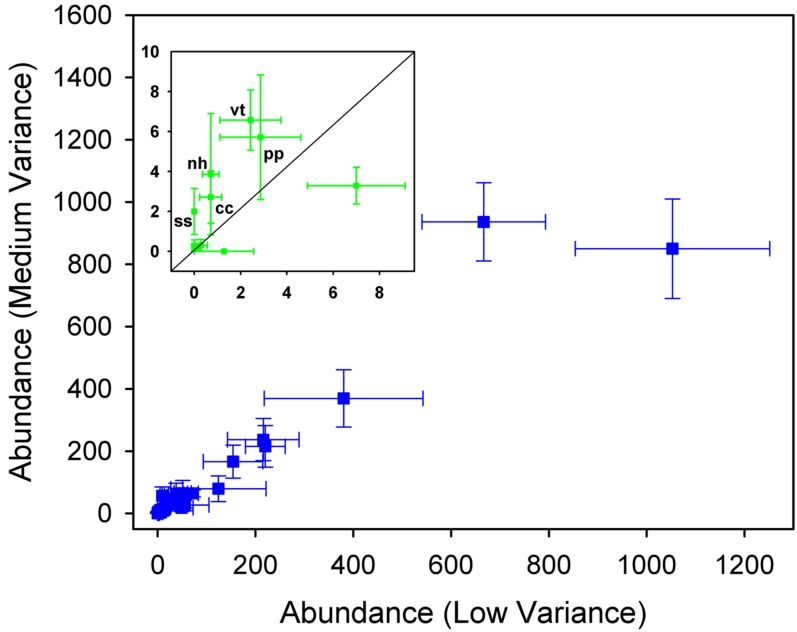
Mean species abundances (±1 s.e.m., n = 18) in Low Variance (horizontal axis) and Medium Variance (vertical axis) treatments. The inset shows an increase in abundance of rare species (mean abundances in the range 0.1–10) associated with enhanced temporal variance of disturbance; cc: *Cystoseira compressa*; nh: *Nemalion helmintoides*; pp: *Padina pavonica*; ss: *Spirorbis* sp.; vt: *Vermetus triqueter*.

Inspection of the rank-abundance curves (Figure 2), however, showed that assemblages in Control and LV conditions had a tail of rare species, represented by singletons, whilst assemblages in MV and HV treatments lacked singletons. Rare species disappeared from HV plots, which had lower species richness compared to the other treatments. In contrast, singletons were missing in the MV condition because rare species entered higher abundance classes under intermediate levels of temporal variance of disturbance. This was the case for 5 of the 29 species (17.2%) in the MV treatment, which were either unobserved or had a lower rank in the LV treatment (Figure 3).

**Figure 4:**
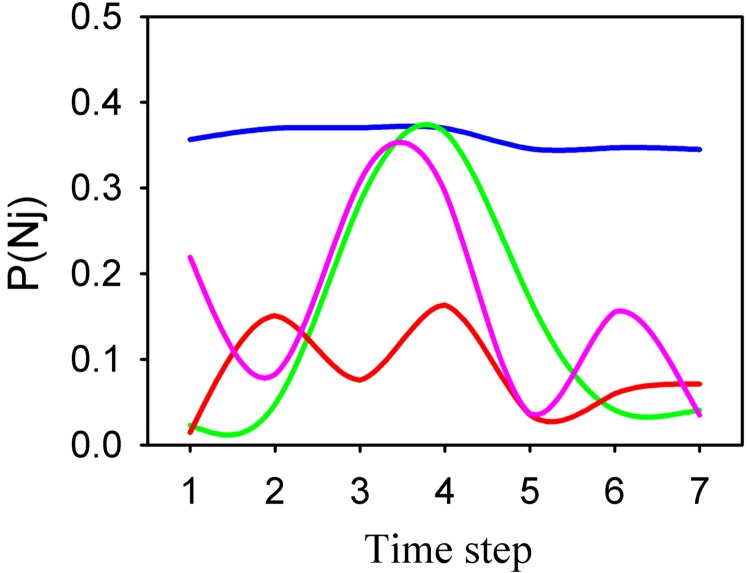
Probabilities of finding singleton species in Control and Experimental plots during the course of the experiment. Parameters θ and *m* were estimated at each time step for each condition. Probabilities were derived from eq. (.5) in Materials and Methods S1, assuming that the relative abundance of singleton species in each local assemblage reflected the relative abundance in the corresponding metacommunity. Blu, green, red and pink lines indicate Control, LV, MV and HV treatments, respectively. Note the lower probability of finding singleton species in the MV treatment and the larger fluctuations in disturbed conditions.

To examine the influence of disturbance on rare species in more detail, we calculated the probability of observing singleton species at every time step during the course of the experiment in the Control, LV, MV and HV conditions using eqn. 18 in [32] (see Materials and Methods S1). This analysis showed that the probability of observing singleton species was much lower in MV than in any other condition during the course of the experiment (Figure 4). This probability also changed considerably through time in LV, MV and HV conditions, whilst it remained fairly constant in the Control.

### Long-term dynamics

Fitting the relative species abundance and species turnover distributions to Control data provided an estimate of *τ* (±1 s.e.) of 16.6 (4.6) years, in agreement with the time required for one turnover determined from field observations of disturbance-induced mortality. Values of *τ* for the LV, MV and HV treatments were 12 (1.9), 2.9 (0.9) and 8 (1.7), respectively. We found values of *b/D* (±1 s.e.) for Control, LV, MV and HV to be 0.258 (0.061), 0.379 (0.0485), 0.651 (0.101) and 0.772 (0.110), respectively. Finally, the parameter *Dτ* (±1 s.e.) was estimated to be 1496.9 (748.7), 114.2 (32.2), 57.8 (16.3) and 57.8 (11.1) for Control, LV, MV and HV conditions, respectively.

## Discussion

The observational data generally supported neutral drift as the mechanism driving temporal changes in assemblages, when variation was quantified with dissimilarity measures that emphasized changes in abundant species. Possible exceptions were the overestimation by the neutral model of observed temporal autocorrelation at the shortest time scale (3 months) and a slight underestimation of observed correlation at time lags 4 and 5. Neutral drift was also supported at the shortest time scale of 3 months and at scales larger than 24 months when variation in rare species was considered. At intermediate time scales (between 3 and 24 months), neutral theory predicted too low values of correlation for dissimilarity measures that emphasized changes in abundance of rare species. The experimental results indicated that a change in temporal variance of disturbance alone – i.e. with no concomitant changes in overall intensity – had no statistically detectable effect on species rank-abundance distributions, so that neutrality was not rejected by these tests. Increasing temporal variance of disturbance, however, led to the disappearance from the MV and HV curves of the tail of rare species that characterized the rank-abundance curves of the Control and LV conditions. The analysis of temporal dynamics indicated that these effects involved density-dependent processes leading to a rare species advantage with increasing levels of environmental fluctuations.

Observed patterns of temporal decay of similarity were consistent with neutrality at most time scales. Ecological drift, however, generated too much short-term temporal variation in abundance of rare species compared to natural patterns, at time scales between 3 months and two years. Increasing intensity of disturbance (regimes of disturbance corresponding to two turnovers of assemblages in Figure S1) emphasized the difference between observed and simulated data. Low autocorrelation was expected under neutral drift where rare species disappeared as a consequence of dispersal limitation (*m* = 0.017). In contrast, a variety of physical and biological processes might have introduced temporal autocorrelation in the dynamics of rare species under natural settings [15]. There is strong evidence that species can track autocorrelation in environmental forcing variables and networks of species interactions – including indirect and facilitative effects – might have contributed to reduce the turnover of rare species [12], [14], [33].

One might argue that higher levels of autocorrelation in observed compared to simulated data reflected a sampling artefact due to the accumulation of many small samples over a long period [34]. Resampling the local assemblage would result in a dilution effect in which the relative abundance of sparse species decreases as new samples are added, producing an excess of rare species. We argue that this effect was unimportant in our study, otherwise the Bray-Curtis and Squared Cord Distance indexes should have produced distinct temporal patterns of decay in similarity for empirical data, as observed with the simulated data. This difference did not occur (see legend of Figure 1); rather, common and rare species underwent the same patterns of temporal change in real assemblages, the latter differing more than the former from neutral expectations.

This situation is similar to what reported by McGill *et al.* [3], who used different similarity measures to compare temporal dynamics of fossil records with expectations based on neutral drift. Although these authors worked at very different temporal scales of those addressed in the present paper, they showed greater inertia (less variability) in empirical compared to simulated data, consistently across similarity measures. In a spatial context, Condit *et al.* [10] found adequate fit of a neutral model to empirical estimates of beta diversity in tropical forests at scales between 0.2 and 50 km. Beyond these scales, however, the model overestimated spatial turnover in species composition. There is, therefore, evidence that neutral models can fit empirical data at some scales, but not at others. Our results add further complexity to this view, indicating that the fit of neutral models to observed patterns of variation in assemblages may depend on the relative contribution of rare and common species to temporal (spatial) dynamics and the level of disturbance considered (Figure S1).

Collectively, the data in the literature and our findings suggested that neutrality may be more common at intermediate spatial and temporal scales [20], [35]. Why this should be so? It has been shown that the neutral theory predicts unrealistically high rates of species turnover over evolutionary time and this can explain the discrepancy between empirical and modeled data at these very large temporal scales [3], [36]. Environmental heterogeneity is invoked as an explanation for deviations between empirical patterns and neutral expectations in the spatial context and over ecological time [7], [9], [10]. We propose that environmental heterogeneity can either mask or emphasize differences between neutral and non-neutral dynamics, depending on choices of sampling design and analytical approaches. Non-neutral species responses to environmental heterogeneity are likely to be revealed in studies that examine variation in assemblages continuously in space or time [3], [10]. Neutral responses, in contrast, are likely to emerge when samples collected over broad spatial and temporal scales are pooled in a single analysis. If the sampling design embraces a wide range of environmental conditions, species are likely to be sampled both in favourable and unfavourable habitats so that interspecific differences in ecological requirements are averaged out. The extent to which this averaging effect may affect conclusions about species neutrality with respect to environmental heterogeneity remains unclear.

The field experiment did not reject neutrality at the time scale of two years. The rank-abundance distributions obtained under the different perturbation regimes were all fitted by the most parsimonious neutral model (the mZSM distribution), consistently with the hypothesis of neutrality. Although these distributions did not differ among experimental conditions, species that were rare in the LV treatment were affected positively by an increase of temporal variance of disturbance (MV condition). Thus, rare species benefited from an initial increase in environmental variance above ambient levels, although some of these species could not persist under the most extreme regime of temporal variance of disturbance (HV condition).

We recognize that observing no response to an experimental manipulation is not necessarily evidence that the null hypothesis is correct. Hence, observing no significant variation in rank-abundance distributions to changes in environmental variability may not necessarily be evidence of neutrality. Theoretical studies, for example, have reported no effect of the temporal characteristics of the disturbance regime on long-term outcomes of non-neutral assemblages [47], [48]. Whether non-neutral assemblages are also resistant to changes in temporal variance of disturbance at the temporal scale of two years (the duration of the experiment) remains to be assessed. Clearly, the inability to associate measures of precision to fitted rank-abundance distributions and the lack of a well defined procedure to compare these curves across experimental conditions are objective limitations to strong inferential tests [11], [38].

As an attempt to mitigate these problems, we compared observed values of the LR statistic to null distributions originated by simulating neutral drift under the same levels of species richness and similar disturbance regimes as those obtained in Control and manipulated conditions. Despite difficulties in interpretation, our experimental results could explain why, in the correlative analysis, neutral dynamics deviated from observed patterns more when the similarity measure emphasized changes in abundance of rare species than when reflecting changes in common species. Abundant specie appeared insensible to changes in temporal variance of disturbance and their long-term dynamics were described well by neutral drift. Rare species, in contrast, were affected by changes in temporal variance of disturbance and although these effects did not elicit significant changes in the models used to fit the rank-abundance distributions, they may account for the deviations from neutral drift observed under natural settings. Rare species appeared to play a key role in driving temporal changes in assemblages because of their susceptibility to fluctuations in environmental conditions.

Insights into the mechanisms underlying these patterns were provided by the *b/D* parameter, which controls the abundance distribution of rare species [31]. The estimated values of this parameter increased under the more variable regimes of disturbance, indicating that density-dependent processes became relatively more important than demographic stochasticity in a fluctuating environment. Collectively, our findings indicated that temporal variance of disturbance affected the temporal dynamics of assemblages by inducing a density-dependent advantage in rare species [31], [39], [40]. Our results also showed that assemblages exposed to intermediate and, to a lesser extent, high levels of temporal variance of disturbance had greater resilience (i.e. recovery capabilities) than control and LV assemblages.

It should be noted that the analytical expressions used to determine the probability of observing singleton species and to fit the dynamical model of [31] (see Materials and Methods S1) assume that assemblages are at or near stationarity. This assumption is likely to be violated for our disturbed assemblages. Nevertheless, assemblages exposed to different levels of temporal variance of disturbance were likely to deviate from stationarity in similar ways, because intensity of disturbance was constant across treatments. Furthermore, there was good agreement between the empirical estimate of assemblage turnover based on field measurements of disturbance and recovery [41] and the turnover estimated by the model for Control data. Thus, fitting these models to the experimental data should be viewed as a heuristic to assess relative differences in the dynamical properties of assemblages exposed to increasing levels of environmental variance. Future studies should assess the extent to which the models are sensible to deviations from the assumption of stationarity and the development of more realistic non-stationary models is desirable.

Our results have several important ecological implications. First, they offer a unified view of currently separated theories relating environmental variance, density-dependent processes and assemblage dynamics [31], [39], [40]. Our data show that rare species can play a key role in driving assemblage dynamics in fluctuating environments through density-dependent regulation. Second, the susceptibility of rare species to environmental heterogeneity can explain why neutral drift did not match the observed patterns of temporal decay in similarity based on rare species for the first 3–24 months. This outcome suggests that the transitions between non-neutral and neutral dynamics documented in the literature may underscore the spatial or temporal scales at which environmental variance becomes irrelevant to the dynamics of rare species. Finally, the positive response of rare species to temporal variance of disturbance suggests that currently sparse populations may increase in abundance with increasing levels of environmental variability. Rare species can therefore be expected to play a major role in driving assemblage dynamics under climate change, where environmental variability is predicted to increase further in the near future [49].

## Materials and Methods

### Observational data: temporal changes in assemblages

Assemblages were sampled non-destructively with visual counts every three months between 1991 and 2006. Data were obtained from quadrats distributed on a rocky shore about 1 km long in the northwest Mediterranean (43°30′N, 10°20′E). On average, 96 plots were sampled in each occasion, actual numbers varying in relation to weather conditions and availability of resources. Quadrats ranged in size from 10×10 to 20×20 cm, to comply with the requirements of other research projects. These sizes were appropriate to sample the small species of algae and invertebrates that characterized the shore and did not result in different estimates of either mean abundances or spatial and temporal variances of species [50], [51]. About 70% of the data came from newly established random quadrats at each sampling occasion. The remaining observations were collected from permanently marked quadrats that served as controls in other experiments. Estimates of species abundances were obtained by counting all visible organisms in quadrats. For most species of algae individual thalli could not be resolved in the field, so abundances were recorded as counts over 100 points in these cases. Total counts were not constrained to 100 tough, because assemblages were multilayered. Previous studies have shown that fitting neutral models to frequency data generated similar results as those obtained from fits based on individual counts and that the procedure retains its validity for comparative purposes [35], [51]. There were only two species of gastropods in our datasets for which abundances were expressed as counts (this also applies to the experimental data indicated below). Removing these species from the analyses did not affect the results.

Data from the first year of sampling were used to estimate the parameters of the neutral theory by fitting a local Zero-Sum-Multinomial distribution [2], [53]. These parameters were used to simulate ecological drift under neutrality for 60 time steps, corresponding to the following 15 years of observations. At each time step, disturbance killed a number of individuals determined to reflect a complete turnover of assemblages over the period of study, which is a realistic estimate for our system [41]. We also investigated dynamics under levels of mortality corresponding to 0.5 and 2 turnovers in 15 yrs for comparison.

The number of individuals killed at each time step was chosen to reflect seasonal events of disturbance. Simulating neutral drift under constant disturbance may not be realistic, particularly in temperate marine systems where the frequency and intensity of disturbance may vary seasonally. To assess whether there were seasonal patterns of disturbance on our shores, we calculated the number of strong storms (defined as those resulting in waves >3 m high) occurring in the different seasons over the period 2000–2007, for which we could find wave climate data (courtesy of the Istituto Idrografico e Mareografico di Pisa). Over this period, 31% of strong storms occurred in spring, 21% in summer and 24% in each of the two remaining seasons. To incorporate seasonal variation of disturbance in our analysis, we simulated neutral drift by distributing death events across seasons in the same proportion as the observed events of disturbance.

Temporal changes in assemblages were examined with Mantel correlograms and compared between observed and modeled data. This procedure enabled us to determine the temporal scale at which observed patterns of correlation diverged from those predicted by the neutral model. Similarity was quantified using various indexes that emphasized different aspects of assemblage dynamics. We applied the Bray-Curtis index to untransformed data to emphasize changes in the most abundant species, the Jaccard index to emphasize compositional changes and the Square Cord Distance to assess the influence of changes in abundance of rare species [54]. The Bray-Curtis index measuring the dissimilarity between samples *j* and k, was defined as:
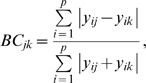
(1)where *y_ij_* and *y_ik_* are the abundances of species *i* (*i* = 1, 2, … *p*) in samples *j* and *k*, respectively. The Jaccard index was defined as:
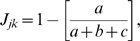
(2)where *a* is the number of species present in both samples *j* and *k*, *b* is the number of species present in sample *j*, but absent in sample *k* and *c* is the number of species present in sample *k*, but absent from sample *j*. This index requires that species which are jointly absent from samples *j* and *k* are first removed. Finally, the Squared Cord Distance index was defined as:
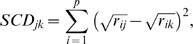
(3)where *r_ij_* and *r_ik_* are the relative abundances of species *i* in samples *j* and *k*, respectively.

Mantel correlograms were generated using package ecodist in R [55]. These analyses assumed second-order (weak) stationarity [54]. This assumption is critical for statistical inference on temporal (or spatial) pattern. Our main purpose was to compare the decay in similarity through time between observed and neutral data using different dissimilarity measures, rather than testing for the significance of Mantel's coefficients to identify particular temporal structures. We based our comparisons on 95% confidence intervals derived by bootstrapping the original data 1000 times with replacement.

Neutral drift was simulated using package untb in R [55]. Estimates of patterns of mortality due to natural disturbance indicated that these assemblages undergo a complete turnover over a period of approximately 15 years [41]. Hence, simulations were run for levels of mortality corresponding to 0.5, 1 and 2 turnovers for each of the similarity indexes (Bray-Curtis, Jaccard and Squared Cord Distance).

### Experimental data: effects of temporal variance of disturbance

To test the sensitivity of assemblages to changes in environmental fluctuations, we manipulated the temporal variance of events of disturbance in 54 experimental plots distributed in the same study area where the long-term observations were collected, between November 2001 and October 2003. Disturbances consisted in the mechanical removal of organisms from experimental plots through a standardized procedure that reproduced the effects of wave shock during heavy storms [56]. The full experiment consisted in a three-factorial design with three levels of temporal variance of disturbance crossed with three levels of intensity and two spatial extents, resulting in 18 treatment combinations. Disturbances consisted in the mechanical removal of organisms from experimental plots of either 50×50 or 100×50 cm. Plots were disturbed using a chisel mounted on a battery drill to mimic the effects of heavy storms. Three independent plots were allocated randomly to each treatment combination. The three levels of intensity of disturbance were generated by scraping the rock surface one, two or three times, respectively. Experimental levels of temporal variance were generated by distributing six events of disturbance more or less evenly over the course of study. Variance was defined with respect to the interval of time (in months) between subsequent disturbances. The lowest level of variance (LV = 2.8 months^2^) was obtained by distributing the events of disturbance at approximately regular intervals. The medium (MV = 8.8 months^2^) and high (HV = 26.8 months^2^) levels of variance were obtained by distributing disturbances more heterogeneously over the course of the study. The experimental levels of intensity, spatial extent and temporal variance of disturbance were realistic for the system under study [56].

Experimental and control plots were sampled seven times during the course of the experiment, with three replicate 12×10 cm quadrats placed randomly in each plot. Only the central 50×50 cm area of the largest plots was sampled, so that abundances were estimated at the same spatial scale in large and small plots. Sampling was done as described for the long-term observational study.

We used species rank-abundance distributions to test the hypothesis that assemblages would retain the same structure under different levels of temporal variability, as expected under neutrality. For these analyses, data for each level of temporal variance of disturbance were combined across levels of intensity and plot size. Given the experimental design, this procedure enabled us to examine the effects of temporal variance of disturbance while keeping intensity and spatial extent constant, so to maximize replication for the main test of interest (*n* = 18). Species rank-abundance distributions were generated by summing the data from the three quadrats in each plot at each sampling occasion and then averaging these sums over the course of the study. Although care is needed when interpreting species rank-abundance distributions obtained from the accumulation of small samples [3], [34], this approach was necessary to examine effects of temporal variance of disturbance properly. Comparisons made at single points in time are meaningless in this kind of experiments, because they confound effects of variance with differences among treatments in the interval of time between sampling and the last event of disturbance (plots allocated to different levels of temporal variance of disturbance were not disturbed at the same time) [57]. Only by considering the average (or total) response of assemblages over the course of the study, could effects of variance be separated from other sources of variation. However, because our data originated from a designed experiment, the species rank-abundance distribution obtained from these data did not differ with respect to sampling intensity or the spatio-temporal scales of sampling and therefore were comparable among treatments.

The meta and local Zero-Sum-Multinomial distributions (mZSM and lZSM, respectively) were fitted to rank-abundance data using Etienne and Ewens sampling formulae, respectively [58]. Model parameters (θ and *m*) were estimated through maximum likelihood, separately for each of the Control, LV, MV and HV conditions. We expected the same model to fit the data from the different treatments equally well if assemblages were insensitive to changes in environmental variance. These tests were based on the likelihood-ratio statistic (LR) obtained by comparing the fit of the two distributions separately to the Control and each of the disturbed conditions. Because the LR statistic is negatively related to species richness under a true null hypothesis of neutrality and species richness differed among assemblages in the experiment, we could not assess the significance of the LR statistic by comparing observed values to a *χ* ^2^ distribution. As an alternative, we assessed the significance of the LR statistic by comparing observed values to null distributions derived by simulating neutral drift under the same levels of richness observed for the Control, LV, MV and HV conditions and under realistic disturbance regimes.

To generate the null distribution for a particular condition, we simulated neutral drift in 10 plots each containing 400 individuals. Data were generated using Etienne's algorithm [58] with parameters θ = 6 and *m* = 0.039, to approximate the parameters estimated from the Control data (with estimates of θ of 4.9 and 7.7 for the mZSM and lZSM distributions, respectively). Plots were disturbed 6 times, corresponding to the number of disturbances imposed in the field experiment. The average disturbance was of 133 deaths of individuals, corresponding to the removal of about one-third of the assemblage, as in the real experiment [56]. Temporal variance of disturbance was imposed by selecting the actual number of individuals to be removed at each time step from a normal distribution with mean 400 individuals and standard deviations set as 5, 10 and 20% of the mean for the LV, MV and HV treatments, respectively. Data were pooled over the 10 plots after each disturbance and pooled assemblages were averaged over the 6 disturbances. Thus, the simulated assemblages resembled the real ones with respect to sample size and the way data were combined across plots and time for the analysis. This procedure was recycled until the final assemblage had the same richness of the particular experimental condition being simulated. When also this condition was met, the data were analyzed by fitting the lZSM and mZSM distributions and the LR statistic was calculated. The procedure was repeated 999 times for each of the LV, MV and HV treatments. The same was done for the Control condition, but in this case there was no variance in disturbance and 9 individuals were killed at each time step. In this way a cycle of 6 disturbances resulted in 54 deaths that corresponded to about two years of drift (assuming that one turnover would have occurred in 15 years), similarly to the duration of the experiment. We also performed simulations in which temporal variance of disturbance was generated by sampling a uniform distribution in the range 200–600 deaths, but this did not change the qualitative outcome of the results.

### Long-term dynamics

To test the hypothesis that temporal variance of disturbance affects the long-term dynamics of the system, we fitted the relative species abundance and species turnover distributions to control and experimental data using eqn. 1 and eqn. 2 in Azaele *et al.* [31]. These fits enabled the simultaneous estimation of the parameters *Dτ*, *b/D* and *τ* (details in Materials and Methods S1), where *τ* is the characteristic time scale of species turnover, *b* is a measure of the rate of immigration and accounts for density-dependent effects, while *D* accounts for demographic stochasticity. These analyses enabled us to assess the relative importance of density-dependence and demographic stochasticity under different levels of environmental fluctuations in a controlled experiment.

## Supporting Information

Materials and Methods S1 (0.06 MB DOC)

Figure S1 Mantel correlograms for different regimes of disturbance and similarity indexes. Shown are patterns of temporal decay in similarity for observed (blue or open symbols) and neutral (grey symbols) data. Open symbols are used to indicate Mantel's coefficients for observed data that did not differ significantly from zero. Probabilities were not corrected for multiple testing. Lines around observed data are 95% bootstrapped Confidence Intervals. Bars for neutral data are ±1 s.d. obtained form 100 replicated simulations. The first panel complements the results presented in the main text for a regime of disturbance corresponding to one turnover of assemblages. The other panels compare the different indexes of similarity for patterns of mortality corresponding to 0.5 and 2 turnovers over 15 years. Differences between observed and modeled data based on the SCD (Squared Cord Distance) index were less pronounced under very low levels of mortality (disturbance = 0.5 turnover). In contrast, the degree to which the neutral model based on Jaccard and Bray-Curtis measures overestimated autocorrelation at the smallest time scales, was emphasized by low levels of mortality. Note the different scales on the vertical axes.
(2.47 MB DOC)

Figure S2 Neutral drift under constant and variable regimes of disturbance and immigration. (A) Shown are mean species rank-abundance distributions over 100 replicated simulations (±1 s.d.) of neutral drift under constant (bleu symbol) and variable (pink symbol) regimes of disturbance. Neutral drift was simulated for a local assemblage of 5000 individuals connected to a metacommunity of 100000 individuals, with parameter θ = 7.7 and *m* = 0.039, corresponding to the values estimated from the undisturbed condition in the experiment (see main text). Simulations were run for 100 generations with an average death rate of 500 individuals per time step (corresponding to 10 turnovers of the local assemblage). This was the exact number of individuals removed at each time step under constant disturbance. For the variable regime of disturbance, the actual number of individuals removed at each time step was determined by sampling a normal distribution of μ = 500 and σ^2^ = 2500. Changing values of parameters did not change the qualitative outcome of the simulation, with constant and variable regimes of disturbance yielding overlapping rank-abundance distributions. Other details are explained in Materials and Methods of the main text. (B) As in (A), but with m varying proportionally to the number of individuals removed at each time step (pink symbol), so that the rate of immigration tracks temporal fluctuations in disturbance.
(0.73 MB TIF)

Figure S3 Null distributions of the likelihood-ratio statistic (LR) originated by simulating neutral drift under the same levels of richness observed for the Control, LV, MV and HV conditions.
(1.28 MB TIF)

## References

[1] Maurer BA (1999). Untangling ecological complexity.

[2] Hubbell SP (2001). The Unified Neutral Theory of Biodiversity and Biogeography.

[3] McGill BJ, Hardly EA, Maurer BA (2005). Community inertia of quaternary small mammal assemblages in North America.. Proc Natl Acad Sci USA.

[4] Tilman D (1988). Plant strategies and the dynamics and structure of plant communities.

[5] Chesson P (2000). Mechanisms of maintenance of species diversity.. Annu Rev Ecol Syst.

[6] Harpole WS, Tilman D (2006). Non-neutral patterns of species abundance in grassland communities.. Ecol Lett.

[7] Bell G (2000). The distribution of abundance in neutral communities.. Am Nat.

[8] Tilman D (2004). Niche tradeoffs, neutrality, and community structure: a stochastic theory of resource competition, invasion, and community assembly.. Proc Natl Acad Sci USA.

[9] Maurer BA, McGill BJ (2004). Neutral and non-neutral macroecology.. Basic Appl Ecol.

[10] Condit R, Pitman N, Leigh EG, Chave J, Terborgh J, Foster RB (2002). Beta-diversity in tropical forest trees.. Science.

[11] McGill BJ, Maurer BA, Weiser MD (2006). Empirical evaluation of neutral theory.. Ecology.

[12] Steele JH (1985). A comparison of terrestrial and marine ecological systems.. Nature.

[13] Halley JM (1996). Ecology, evolution and 1/f-noise.. Trends Ecol Evol.

[14] Vasseur A, McCann KS (2007). The impact of environmental variability on ecological systems.

[15] Benedetti-Cecchi L (2007). Neutral theory and 1/f noise make similar predictions of assemblage dynamics.. Trends Ecol Evol.

[16] Etienne RS, Olff H (2005). Bayesian analysis of species abundance data: assessing the relative importance of dispersal and niche-partitioning for the maintenance of biodiversity.. Ecol Lett.

[17] Etienne R (2007). A neutral sampling formula for multiple samples and an exact test for neutrality.. Ecol Lett.

[18] Pueyo S, He F, Zillo T (2007). The maximum entropy formalism and the idiosyncratic theory of biodiversity.. Ecol Lett.

[19] Alonso D, Ostling A, Etienne R (2008). The implicit assumption of symmetry and the species abundance distribution.. Ecol Lett.

[20] Wootton JT (2005). Field parameterization and experimental test of the neutral theory of biodiversity.. Nature.

[21] Chave J (2004). Neutral theory and community ecology.. Ecol Lett.

[22] Alonso D, Etienne RS, McKane AJ (2006). The merits of neutral theory.. Trends Ecol Evol.

[23] Gravel D, Canham DC, Beaudet M, Messier C (2006). Reconciling niche and neutrality.. Ecol Lett.

[24] Holt RD (2006). Emergent neutrality.. Trends Ecol Evol.

[25] Adler PB, HilleRisLambers J, Levine JM (2007). A niche for neutrality.. Ecol Lett.

[26] Cadotte MW (2007). Concurrent niche and neutral processes in the competition-colonization model of species coexistence.. Proc R Soc B.

[27] Alonso D, Etienne RS, McKane AJ (2007). Response to Benedetti-Cecchi: neutrality and environmental fluctuations.. Trends Ecol Evol.

[28] Hu XS, He F, Hubbell SP (2007). Species diversity in local neutral communities.. Am Nat.

[29] Benton TG, Lapsley CT, Beckerman AP (2001). Population synchrony and environmental variation: an experimental demonstration.. Ecol Lett.

[30] Boyce MS, Haridas CV, Lee CT, NCEAS Stochastic Demography Working Group (2006). Demography in an increasingly variable world.. Trends Ecol Evol.

[31] Azaele S, Pigolotti S, Banavar JR, Maritan A (2006). Dynamical evolution of ecosystems.. Nature.

[32] McKane AJ, Alonso D, Solé RV (2004). Analytic solution of Hubbell's model of local community dynamics.. Theor. Pop Biol.

[33] Vasseur DA, Yodzis P (2004). The color of environmental noise.. Ecology.

[34] McGill BJ (2003). Does mother nature really prefer rare species or are log-left-skewed SADs a sampling artefact?. Ecol Lett.

[35] Fargione J, Brown CS, Tilman D (2003). Community assembly and invasion: an experimental test of neutral versus niche processes.. Proc Natl Acad Sci USA.

[36] Ricklefs RE (2003). A comment on Hubbell's zero-sum ecological drift model.. Oikos.

[37] Magurran AE (2004). Measuring biological diversity.

[38] McGill BJ, Etienne RS, Gray J, Alonso D, Anderson MJ (2007). Species abundance distributions: moving beyond single prediction theories to integration within an ecological framework.. Ecol Lett.

[39] Chesson PL, Warner RR (1981). Environmental variability promotes coexistence in lottery competitive systems.. Am Nat.

[40] Volkov I, Banavar JR, He F, Hubbell SP, Maritan A (2005). Density dependence explains tree species abundance and diversity in tropical forests.. Nature.

[41] Benedetti-Cecchi L (2000). Predicting direct and indirect effects during succession in a midlittoral rocky shore assemblage.. Ecol Monogr.

[42] Magurran AE (2007). Species abundance distributions over time.. Ecol Lett.

[43] Mayo DG (1996). Error and the growth of experimental knowledge.

[44] Ugland KI, Gray JS (1982). Lognormal distributions and the concept of community equilibrium.. Oikos.

[45] Magurran AE, Henderson PA (2003). Explaining the excess of rare species in natural species abundance distribution.. Nature.

[46] Ulrich W, Zalewski M (2006). Abundance and co-occurrence patterns of core and satellite species of ground beetles on small lake islands.. Oikos.

[47] Chesson P, Huntly N (1997). The roles of harsh and fluctuating conditions in the dynamics of ecological communities.. Am Nat.

[48] Wootton JT (1998). Effects of disturbance on species diversity: a multitrophic perspective.. AM Nat.

[49] Easterling DR, Meehl GA, Parmesan C, Changnon SA, Karl TR, Mearns LO (2000). Climate extremes: observations, modeling, and impacts.. Science.

[50] Menconi M, Benedetti-Cecchi L, Cinelli F (1999). Spatial and temporal variability in the distribution of algae and invertebrates on rocky shores in the northwest Mediterranean.. J Exp Mar Biol Ecol.

[51] Benedetti-Cecchi L (2001). Variability in abundance of algae and invertebrates at different spatial scales on rocky sea shores.. Mar Ecol Prog Ser.

[52] Connolly SR, Hughes TP, Bellwood DR, Karlson RH (2005). Community structure of corals and reef fishes at multiple scales.. Science.

[53] Alonso D, McKane AJ (2004). Sampling Hubbell's neutral theory of biodiversity.. Ecol Lett.

[54] Legendre P, Legendre L (1998). Numerical Ecology.

[55] R Development Core Team (2004). R: A Language and Environment for Statistical Computing.

[56] Bertocci I, Maggi E, Vaselli S, Benedetti-Cecchi L (2005). Contrasting effects of mean intensity and temporal variation of disturbance on assemblages of rocky shores.. Ecology.

[57] Benedetti-Cecchi L (2003). The importance of the variance around the mean effect size of ecological processes.. Ecology.

[58] Etienne RS (2005). A new sampling formula for neutral biodiversity.. Ecol Lett.

[59] Burnham KP, Anderson DR (1998). Model Selection and Inference: A practical Information-Theoretic Approach.

[60] Fisher R, Corbet AS, Williams CB (1943). The relation between the number of species and the number of individuals in a random sample of an animal population.. J Anim Ecol.

[61] Bulmer MG (1974). On fitting the Poisson Lognormal Distribution to species-abundance data.. Biometrics.

[62] Dornelas M, Connolly SR, Hughes TP (2006). Coral reef diversity refutes the neutral theory of biodiversity.. Nature.

