# Supplementary material for: Neutrality and the Response of Rare Species to Environmental Variance
Source: PLoS One. 2008 Jul 23;3(7):e2777. doi: 10.1371/journal.pone.0002777 (PMC2481292; doi:10.1371/journal.pone.0002777)
Supplement: Materials and Methods S1 — (0.06 MB DOC) [file pone.0002777.s001.doc]

Supporting information

Materials and Methods S1

## Neutrality and the response of rare species to environmental variance

## Lisandro Benedetti-Cecchi^1^, Iacopo Bertocci^1^, Stefano Vaselli^2^, Elena Maggi^1^, Fabio Bulleri^1^

^1^Dipartimento di Biologia, Università di Pisa, Pisa, Italy

^2^Centre for Marine and Environmental Research, Laboratory of Coastal Biodiversity, University of Port, Portugal

**Corresponding Author:**

Lisandro Benedetti-Cecchi, Dipartimento di Biologia,University of Pisa, Via Derna 1, I-56126 Pisa, Italy, Tel. +39 050 2211413, Fax: +39 050 2211410, e-mail: lbenedetti@biologia.unipi.it

**Table of Contents:**

1. Relative species abundance and species turnover distributions
2. Probability of observing singleton species
3. References

**1. Relative species abundance and species turnover distributions**

Temporal dynamics were examined using the approach described in Azaele *et al.* [2]. The three parameters *Dτ*, *b/D* and *τ* were estimated by fitting the relative species abundance and species turnover distributions to experimental data using the least-squares method. The relative species abundance distribution is defined as:

$P_{\text{RSA}}(x)=\frac{(D\tau)^{-b/D}}{\Gamma\left(b/D\right)}x^{b/D-1}e^{-x/D\tau}$, (.1)

where *τ* is the characteristic time scale of species turnover, *b* is a measure of the rate of immigration and accounts for density-dependent effects, *D* accounts for demographic stochasticity and Γ is the Gamma function.

The species turnover distribution is defined as

$P_{\text{STD}}(\lambda,t)=A\frac{(\lambda+1)}{\lambda}\frac{(e^{t/\tau})^{b/2D}}{1-e^{-t/\tau}}\frac{\sinh(t/2\tau)}{\lambda}^{b/D+1}\left(\frac{4\lambda^{2}}{(\lambda+1)^{2}e^{t/\tau}-4\lambda}\right)^{b/D+0.5}$, (.2)

where PSTD(*λ*,*t*) is the probability that the ratio of the abundances of a species separated by a time interval *t*, x(*t*)/x(0), is equal to *λ* under stationary conditions and A is the normalization constant:

$A=\frac{2^{b/D-1}}{\sqrt{\pi}}\frac{\Gamma(b/D+0.5)}{\Gamma(b/D)}$, (.3)

Parameters were estimated by fitting eq. (.1) and eq. (.2) to species abundance and dynamical data, respectively and minimizing

$\chi^{2}(b/D,D\tau,\tau)=\frac{\chi_{RSA}^{\min^{2}}}{\chi_{STD}^{\min^{2}}}\chi_{STD}^{2}\left(\frac{b}{D},\tau\right)+\chi_{RSA}^{2}\left(\frac{b}{D},D\tau\right)$, (.4)

where$\chi_{RSA}^{\min^{2}}$is the minimum normalized χ^2^ with respect to eq. (.1) and$\chi_{STD}^{\min^{2}}$is the minimum normalized χ^2^ with respect to eq. (.2).

**2. Probability of observing singleton species**

We used eq. (18) in McKane *et al.* [3] to define the probability *P_s_*(*n;J;m;x*) of finding a species with *n* individuals in a local assemblage of size *J* with relative abundance *x* in the metacommunity to which it is connected through the immigration parameter *m*. The analytical solution for *P_s_* is given as,

$P_{s}(n,J,m,x)=\left(\frac{J}{n}\right)\frac{\Gamma(n+\gamma x)}{\Gamma(\gamma x)}\frac{\Gamma(\nu-m)}{\Gamma(\nu-J)}\frac{\Gamma(\lambda+\nu-J)}{\Gamma(\lambda+\nu)}$, (.5)

where Γ is the Gamma function and:

$\lambda=\gamma x$,

$\nu=J+\gamma(1-x)$,

$\lambda=\frac{m(J-1)}{1-m}$, (.6)

## References

1. Hubbell SP (2001) *The Unified Neutral Theory of Biodiversity and Biogeography*. Princeton Univ. Press, Princeton, NJ. 375 p.
2. Azaele S, Pigolotti S, Banavar JR, Maritan, A (2006) Dynamical evolution of ecosystems. Nature 444:926-928.
3. McKane AJ, Alonso D, Solé RV (2004) Analytic solution of Hubbell’s model of local community dynamics. Theor Popul Biol 65:67-73.
