# Supplementary figures and images for: Neutrality and the Response of Rare Species to Environmental Variance
Source: PLoS One. 2008 Jul 23;3(7):e2777. doi: 10.1371/journal.pone.0002777 (PMC2481292; doi:10.1371/journal.pone.0002777)

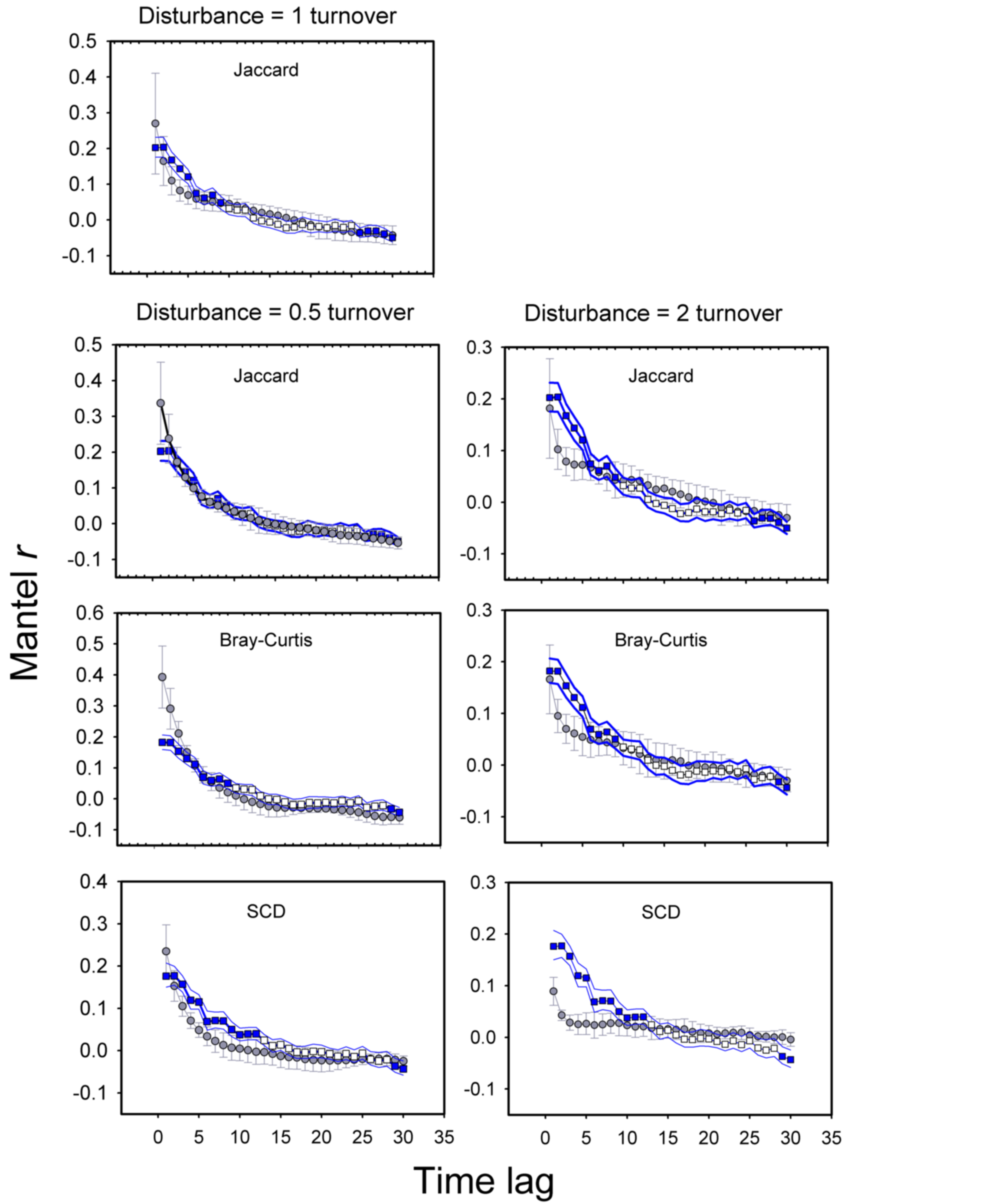

Supplement: Figure S1 — Mantel correlograms for different regimes of disturbance and similarity indexes. Shown are patterns of temporal decay in similarity for observed (blue or open symbols) and neutral (grey symbols) data. Open symbols are used to indicate Mantel's coefficients for observed data that did not differ significantly from zero. Probabilities were not corrected for multiple testing. Lines around observed data are 95% bootstrapped Confidence Intervals. Bars for neutral data are ±1 s.d. obtained form 100 replicated simulations. The first panel complements the results presented in the main text for a regime of disturbance corresponding to one turnover of assemblages. The other panels compare the different indexes of similarity for patterns of mortality corresponding to 0.5 and 2 turnovers over 15 years. Differences between observed and modeled data based on the SCD (Squared Cord Distance) index were less pronounced under very low levels of mortality (disturbance = 0.5 turnover). In contrast, the degree to which the neutral model based on Jaccard and Bray-Curtis measures overestimated autocorrelation at the smallest time scales, was emphasized by low levels of mortality. Note the different scales on the vertical axes. (2.47 MB DOC) [file pone.0002777.s002.tif]

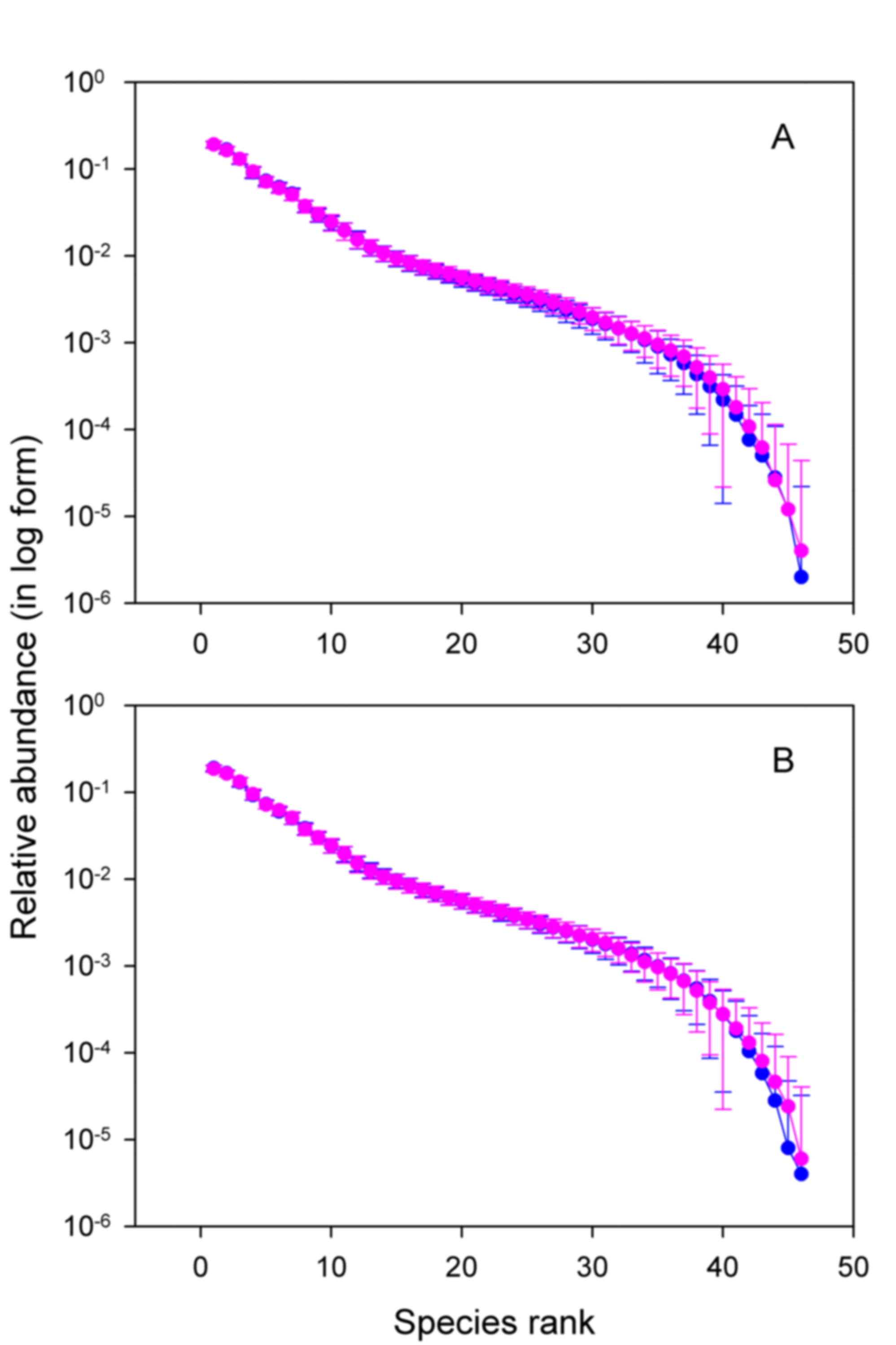

Supplement: Figure S2 — Neutral drift under constant and variable regimes of disturbance and immigration. (A) Shown are mean species rank-abundance distributions over 100 replicated simulations (±1 s.d.) of neutral drift under constant (bleu symbol) and variable (pink symbol) regimes of disturbance. Neutral drift was simulated for a local assemblage of 5000 individuals connected to a metacommunity of 100000 individuals, with parameter θ = 7.7 and m = 0.039, corresponding to the values estimated from the undisturbed condition in the experiment (see main text). Simulations were run for 100 generations with an average death rate of 500 individuals per time step (corresponding to 10 turnovers of the local assemblage). This was the exact number of individuals removed at each time step under constant disturbance. For the variable regime of disturbance, the actual number of individuals removed at each time step was determined by sampling a normal distribution of μ = 500 and σ2 = 2500. Changing values of parameters did not change the qualitative outcome of the simulation, with constant and variable regimes of disturbance yielding overlapping rank-abundance distributions. Other details are explained in Materials and Methods of the main text. (B) As in (A), but with m varying proportionally to the number of individuals removed at each time step (pink symbol), so that the rate of immigration tracks temporal fluctuations in disturbance. (0.73 MB TIF) [file pone.0002777.s003.tif]

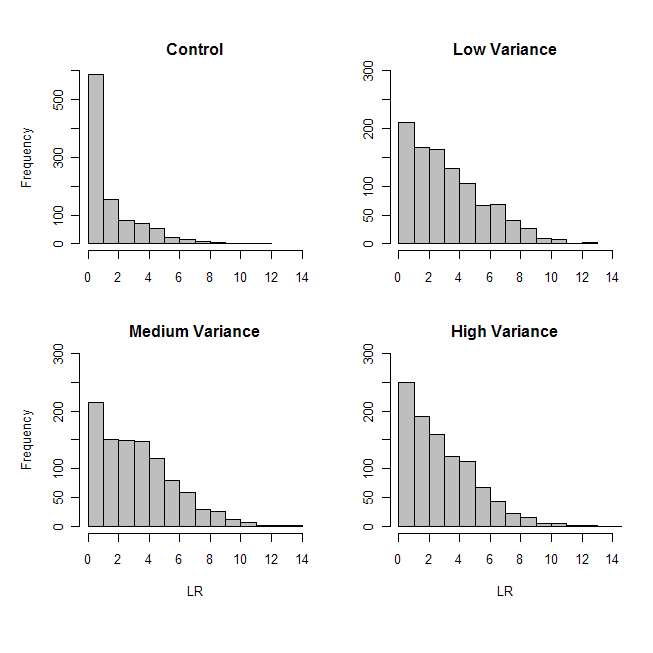

Supplement: Figure S3 — Null distributions of the likelihood-ratio statistic (LR) originated by simulating neutral drift under the same levels of richness observed for the Control, LV, MV and HV conditions. (1.28 MB TIF) [file pone.0002777.s004.tif]
